# Antiviral Activities of Silymarin and Derivatives

**DOI:** 10.3390/molecules24081552

**Published:** 2019-04-19

**Authors:** Ching-Hsuan Liu, Alagie Jassey, Hsin-Ya Hsu, Liang-Tzung Lin

**Affiliations:** 1 Graduate Institute of Medical Sciences, College of Medicine, Taipei Medical University, Taipei 110, Taiwan; julia.chliu@gmail.com; 2 Department of Microbiology & Immunology, Dalhousie University, Halifax, NS B3H 4R2, Canada; 3 International Ph.D. Program in Medicine, College of Medicine, Taipei Medical University, Taipei 110, Taiwan; alagie_jassey@yahoo.com; 4 Department of Microbiology and Immunology, School of Medicine, College of Medicine, Taipei Medical University, Taipei 110, Taiwan; jg930727@gmail.com

**Keywords:** silymarin, flavonolignans, antiviral, drug development

## Abstract

Silymarin flavonolignans are well-known agents that typically possess antioxidative, anti-inflammatory, and hepatoprotective functions. Recent studies have also documented the antiviral activities of silymarin and its derivatives against several viruses, including the flaviviruses (hepatitis C virus and dengue virus), togaviruses (Chikungunya virus and Mayaro virus), influenza virus, human immunodeficiency virus, and hepatitis B virus. This review will describe some of the latest preclinical and clinical studies detailing the antiviral profiles of silymarin and its derivatives, and discuss their relevance for antiviral drug development.

## 1. Silymarin, Its Components, and Derivatives

Silymarin, an extract from the seed of the milk thistle plant (*Silybum marianum* [*S. marianum*]) is widely known for its hepatoprotective functions, mainly due to its anti-oxidative, anti-inflammatory, and immunomodulatory effects [1]. The primary bioactive components of the extract consist of several flavonolignans (silybin, silychristin, silydianin, isosilybin, and dehydrosilybin), and a few flavonoids, mainly taxifolin [2]. The mixture of silybin A and silybin B (1:1) is also known as silibinin (C_25_H_22_O_10_, PubChem CID: 31553; Figure 1), which makes up the major active ingredient (roughly 50%) of silymarin [2,3]. Although silymarin is known mostly for its hepatoprotective functions, accumulating evidence now suggests that the extract possesses potent antiviral activities against numerous viruses, particularly hepatitis C virus (HCV). Consequently, silymarin is the most commonly consumed herbal product among HCV-infected patients in western countries [4]. Despite its potent medicinal effects, silymarin suffers from poor solubility which affects its bioavailability in vivo. To improve the issue, the chemically-hydrophilized silibinin, Legalon^®^ SIL (C_66_H_56_Na_4_O_32_, PubChem CID: 76956344), was developed by the pharmaceutical company Rottapharm Madaus (Monza, Italy) for the administration by intravenous infusion, and the drug was further granted orphan medicinal product designation (EU/3/10/828) from the European Medicines Agency (EMA) for the prevention of recurrent hepatitis C in liver transplant recipients in 2010 [5]. To date, silymarin and its derivatives have been examined for potential bioactivities against several viruses and various strategies to address its drug delivery challenges have also been explored. This review examines the current literature concerning the antiviral effects of silymarin and silymarin-derived compounds used in preclinical and clinical studies, the challenges to clinical application, as well as its prospects as clinically applicable antiviral agents.

## 2. Antiviral Activity of Silymarin and Its Derivatives In Vitro, In Silico, and In Vivo

Viral infections represent important public health concern and socioeconomic burden globally. Presently, numerous viral infectious diseases are without effective vaccines and/or specific antiviral treatments. The increased significance of viruses as human pathogens, and the rising epidemic outbreaks worldwide due to increased population density and migration/travel, underscore the need to continuously identify antiviral strategies against these infectious agents. Silymarin and its derivatives have been reported to possess potent antiviral activities against a number of viruses by targeting multiple steps of the viral life cycle. We describe below the antiviral activities of silymarin and its derivatives against different important human viruses in preclinical studies. Results of the in vitro or in silico studies as well as the in vivo studies are summarized in Table 1 and Table 2, respectively.

### 2.1. The Flaviviridae Family

Flaviviruses are (+)ssRNA viruses that include important human pathogens such as hepatitis C virus (HCV) and dengue virus (DENV). HCV is known to cause chronic infection (hepatitis C) that can lead to end-stage liver diseases such as cirrhosis and hepatocellular carcinoma (HCC) [6]. DENV, on the other hand, is the etiologic agent of dengue fever (DF) and the more severe dengue hemorrhagic fever (DHF) and dengue shock syndrome (DSS), which are fatal illnesses that could lead to death in young children [7]. Currently, there are no effective vaccines against these viruses. Although the treatments for HCV infection has remarkably improved with the advent of direct-acting antivirals (DAAs), important issues such as cost, selection of drug-resistant mutants, and challenges in the difficult-to-treat populations have limited the widespread use of these drugs. DENV infection on the other hand, has no available antiviral treatments. These circumstances necessitate the search for novel/alternative forms of therapy to complement the existing treatment options.

#### 2.1.1. Hepatitis C Virus

The effect of silymarin on HCV has been extensively studied and the antiviral activity of the drug against HCV in vitro is well documented. Using a standardized silymarin extract (MK-001), Polyak et al. demonstrated that MK-001 not only inhibited the genotype 2a HCV strain JFH-1 infection in both the pretreatment and post-infection analysis, but also blocked TNF-α and NFκB transcriptional activity in peripheral blood mononuclear cells (PBMCs) and hepatoma Huh-7 cells, respectively, suggesting that the extract possesses both antiviral and anti-inflammatory bioactivities [8]. Further mechanistic studies demonstrated that although MK-001 treatment alone only modestly affected the interferon (IFN) JAK-STAT pathway, the combination of MK-001 with IFN-α augmented the antiviral efficacy of exogenously added IFN, leading to the conclusion that the antiviral effect of MK-001 is mediated by potentiating the JAK-STAT antiviral signaling pathway which, in turn, inhibits HCV replication.

Following the above discovery, the same authors in two independent studies demonstrated that silymarin treatment blocked different steps of the HCV (JFH-1) life cycle, including entry/fusion, replication, and virion production in the host cells [9,10]. Furthermore, silymarin and its derived pure compounds exhibited potent hepatoprotective functions by inhibiting the HCV-induced oxidative stress, NFκB-dependent transcription, and T-cell receptor (TCR)-mediated proliferation [9]. Interestingly, both studies observed an impact of silymarin on HCV NS5B RNA-dependent RNA polymerase (RdRp) activity, albeit at concentrations higher than that required for its anti-HCV effect. Consistent with the inhibition of the RdRp activity, Belkacem et al. employing silybin A, silybin B, and Legalon^®^ SIL demonstrated that all three forms of silymarin components inhibited HCV replication by targeting the HCV RdRp activity of NS5B, with an IC50 value ranging between 75–100 μM [11].

In contrast, Blaising et al. in 2013 showed that the major bioactive component silibinin exerts antiviral effect against HCV by blocking clathrin-mediated endocytosis [12]. Another study in 2013 indicated that silibinin impedes HCV infection by targeting the HCV NS4B protein [13], which is known to mediate the membranous web formation where HCV RNA replication occurs [14] and hence affecting the morphogenesis of the viral replication sites. More recently, we developed silibinin nanoparticles (SB-NPs) and showed that both the SB-NPs and the conventional silibinin inhibited HCV infection by blocking viral cell-to-cell spread [15]. However, in our hand, the drug had minimal impact on other steps of the viral life cycle (entry, replication, and virion production) or in modulating the type I IFN response.

In 2016, DebRoy et al. investigated the antiviral effect of intravenous Legalon^®^ SIL monotherapy on uPA-SCID chimeric mice with humanized livers [16]. Mice chronically infected with HCV were treated with different intravenous doses of Legalon^®^ SIL (469, 265 or 61.5 mg/kg) for 14 days before analyzing serum HCV, human albumin, and liver HCV RNA levels. The results demonstrated that Legalon^®^ SIL monotherapy led to a biphasic serum viral decline without affecting the human albumin levels, suggesting that the antiviral effect observed was not due to a decline in the human hepatocytes. Furthermore, administration of Legalon^®^ SIL induced anti-inflammatory and anti-proliferative gene expressions that were demonstrated by a decrease in TNF-α and NFκB-associated transcriptional activations. Interestingly, the microarray analysis showed that Legalon^®^ SIL treatment inhibited the expression of genes such as interleukin 8 (IL-8), nicotamidine N-methyltransferase (NNMT), and osteopontin/secreted phosphoprotein 1 (SPP1), which are known to facilitate HCV replication, consistent with the inhibition of HCV production. These results suggest that Legalon^®^ SIL could efficiently inhibit HCV production in mice in the absence of adaptive immunity in vivo.

Together, the results discussed above support the robust anti-HCV activity of silymarin and its derivatives, although the underlying anti-HCV mechanism varies substantially from study to study. Possible explanations include discrepancy in the source or type of drug used (e.g., silymarin component, purity, and extraction method), variation in experimental design such as concentration and treatment protocol, and the specific model systems employed. This is supported by Wagoner et al.’s work demonstrating that the oral and intravenous (i.e., Legalon^®^ SIL) formulations of silibinin exert different effects on HCV life cycle, inflammation, and antiviral signaling in vitro [17]. Thus, concerted efforts should be made to delineate the main mechanism of action of this promising drug. Nonetheless, given that silymarin and its constituents are bioactive against several cell physiological processes, it is likely that silymarin and its major active components may exhibit impact on multiple steps of the virus life cycle, either directly or indirectly.

#### 2.1.2. Dengue Virus

Using in silico drug development approach against the dengue nonstructural protein 4B (NS4B), Qaddir et al. docked 2750 phytochemicals from different medicinal plants to the DENV NS4B protein and identified nine phytochemicals possessing potential inhibitory effects against NS4B, including silibinin and other compounds from the plant *S. marianum* [18]. This result suggests that silibinin could potentially inhibit DENV viral replication. Given that molecular docking analysis is only predictive, further in vitro and in vivo studies would be necessary to confirm the antiviral effect.

### 2.2. Influenza A Virus

Influenza A virus (IAV) is a highly contagious virus and a leading cause of mortality and morbidity globally. Influenza epidemics and pandemics pose a serious threat to both human and animal populations. Although effective vaccines are available against IAV, these vaccines must be regularly updated due to the ability of the virus to induce frequent antigenic drift and occasional antigenic shifts to its envelope glycoproteins. Moreover, only few IAV antiviral therapeutics have been clinically approved and currently in use including neuraminidase inhibitors [19] and the more recent inhibitor of cap-dependent endonuclease [20]. Therefore, continuous identification of novel therapeutic strategies to expand or complement the existing options against this important pathogen is highly envisaged. Gazák et al. developed silibinin derivatives that were conjugated with long-chain fatty acids and demonstrated their superior anti-influenza virus activity compared to conventional silibinin in plaque reduction assay [21]. Later, using cytopathic effect (CPE) reduction method, Song et al. explored the antiviral activity of silymarin against IAV [22]. The authors demonstrated that silymarin dose-dependently inhibited IAV replication without significant cytotoxicity. Further examination revealed that the silymarin-mediated inhibition of influenza replication occurred through inhibition of late mRNA synthesis. However, whether or not silymarin could modulate other phases of the influenza life cycle was not investigated. The other study that looked at the anti-influenza activity of silymarin was the study by Dai et al. [23]. Due to the importance of autophagy in promoting influenza replication, these authors elegantly designed a bimolecular fluorescence complementation-fluorescence resonance energy transfer (BiFC-FRET) assay to analyze the anti-influenza activity of 89 medicinal plants and discovered that *S. marianum L*. possessed excellent activity in the assay. In order to improve the anti-influenza activity, the authors synthesized five silybin amino acid derivatives (S0–S5) and demonstrated using the sulforhodamine B (SRB) antiviral assay that S3 is the most effective against IAV. Further analysis with plaque inhibition assays revealed that in addition to inhibiting the autophagy elongation complex formation, the S0 and S3 derivatives robustly inhibited IAV replication as well as several physiological processes induced by influenza replication, such as oxidative stress and the activation of extracellular signal-regulated kinase (ERK)/p38 mitogen-activated protein kinase (MAPK) and I kappa B (IκB) kinase (IKK) pathways. In agreement with the report above, the authors demonstrated through a time-course analysis that S0 and S3 mainly inhibited IAV replication without exerting any significant effect on viral adsorption. Therefore, it appears that silymarin and its derivatives’ antiviral activity against IAV is chiefly mediated by the inhibition of viral replication. Finally, in vivo oral administration of S0 and S3 not only increased the survival rate in mice infected with a lethal dose of IAV, but also decreased the viral titers in their lungs [23]. Interestingly, this finding is compatible with the accumulation of free silibinin in lung after oral administration that others and we have observed [15,24]. These results, together, point to a prominent role of silymarin as a potent IAV inhibitor.

### 2.3. Human Immunodeficiency Virus

The human immunodeficiency virus (HIV) is a lentivirus that causes acquired immunodeficiency syndrome. HIV is estimated to currently infect over 38 million people, the majority of whom live in sub-Saharan Africa. The advent of the highly active antiretroviral therapy has monumentally improved the survival rate of the HIV-infected population by substantially decreasing the viral load while at the same time preserving the CD4 count. Despite this advancement, the treatment is life-long and associated with side effects. Currently, there is no effective vaccine against this deadly human pathogen. Therefore, identifying novel therapeutics to complement the existing ones would be expected to further improve the management of HIV-infected patients. Interestingly, about 30% of the HIV-infected patients in Europe and North America are co-infected with HCV [25]. With the aim of identifying drugs that could simultaneously target both HCV and HIV, McClure et al. explored the anti-HIV activity of Legalon^®^ SIL [26]. The authors showed that Legalon^®^ SIL inhibited HIV replication in the HeLa cell line TZM-bl, PBMCs, and the human T lymphoblastic leukemia cell line CME in vitro. Mechanistic studies revealed that Legalon^®^ SIL blocked HIV replication by attenuating cellular functions implicated in T-cell activation and proliferation, hence resulting in fewer CD4+ T cells expressing the HIV co-receptors, CXCR4 and CCR5. In a separate study, the authors further characterized the role of Legalon^®^ SIL in HIV infection and demonstrated that Legalon^®^ SIL treatment at the time of virus adsorption in PBMCs and CEM cells blocked HIV infection [27]. Intriguingly, the authors showed that in contrast to their previous report, silibinin’s perturbation of T-cell metabolism is not involved in its ability to block HIV entry. Thus, it appears that silibinin could simultaneously block HIV entry and T-cell activation. Combined together, these results provide evidence for the robust anti-HIV role of silibinin, which therefore merits further evaluation for potential development as an anti-HIV agent.

### 2.4. The Togaviridae Family

Togaviruses are arthropod-borne (+)ssRNA viruses that contribute to various human diseases. Presently, there is no effective treatment or preventive vaccines against important togaviruses including Chikungunya virus (CHIKV) and Mayaro virus (MAYV). Both CHIKV and MAYV belong to the alphavirus genus and are implicated in a variety of human illnesses such as encephalitis, arthralgia, fever, and rash. Therefore, exploring candidate agents capable of inhibiting these togaviruses may provide potential treatment options.

In 2015, Lani et al. investigated the antiviral effect of several flavonoids including silymarin against CHIKV using a CPE reduction assay and RT-PCR analysis [28]. The authors discovered that silymarin robustly inhibited CHIKV-induced CPE. Mechanistic studies further demonstrated that silymarin inhibited CHIKV infection by targeting the post-entry steps of the viral life cycle.

In a similar study, Camini et al. explored the antiviral activity of silymarin against the related togavirus, MAYV [29]. Using an analogous approach, the CPE reduction assay, the authors demonstrated that silymarin at non-cytotoxic concentrations inhibited MAYV replication. Further experiments demonstrated that silymarin pretreatment inhibited MAYV-induced oxidative stress. However, whether the inhibition of the oxidative stress is due, in part, to the inhibition of viral replication or whether inhibition of reactive oxygen species (ROS) itself is sufficient to impede MAYV replication was not addressed. Nonetheless, the findings above suggest that silymarin mainly inhibit alphaviruses by hindering viral replication.

### 2.5. Hepatitis B Virus

Hepatitis B Virus (HBV) is an important human liver pathogen belonging to the *Hepadnaviridae* family. The virus is estimated to chronically infect 240 million people worldwide killing approximately 1 million people every year due to the HBV-associated end-stage liver diseases such as cirrhosis and HCC [30]. Although effective vaccines against the virus have been in existence for the past few decades, the current treatment strategies can only control and suppress the HBV viral load but unable to cure. Thus, the continuous identification of new treatment strategies against the liver pathogen is still needed. Recently, Umetsu et al. demonstrated that similar to HCV, silibinin inhibited HBV entry into the permissive HepG2-NTCP-C4 and PXB cells by blocking clathrin-mediated endocytosis without affecting HBV-receptor interaction, replication or release [31]. More importantly, the combination of silibinin and Entecavir, a known nucleoside reverse transcriptase inhibitor, reduced HBV DNA in the culture supernatant more than either mono-treatment alone in HepG2-NTCP-C4 cells already established with HBV infection, thus highlighting the anti-HBV potential of silibinin.

In 2008, using a different approach, Wu et al. tested the effect of the silymarin on HBV X protein (HBx) transgenic mice and demonstrated that the natural product possesses therapeutic effects at the early stages of HCC development when given orally to 4–6 weeks old transgenic mice [32]. Specifically, oral administration of silymarin dose-dependently reversed fatty liver changes and restored normal liver histopathology in these animals. Further analysis revealed that administration of silymarin to the precancerous HBx transgenic mice prevented the development of HCC. In contrast, silymarin treatment could not block the progression of established cancer in mice and had no significant effect on the HBx gene expression. The fact that silymarin did not modulate HBx gene expression could imply that the drug does not affect HBV replication, which is consistent with the in vitro study above. Thus, it appears that silymarin blocks HBV infectivity by influencing early viral entry.

In summary, the in vitro and in silico studies described above identify silymarin and its derivatives as attractive antiviral candidates against multiple viruses. The extract or molecular components appear to inhibit viral infection by targeting several steps of the viral life cycle either directly or indirectly, thereby highlighting the robust antiviral activities of silymarin and its derivatives.

## 3. Antiviral Activity of Silymarin and Its Derivatives in Clinical Trials 

To date, clinical studies of silymarin, its component, and their derivatives are mostly limited to HCV-related infections due to their pronounced effect in preclinical studies. Here we review the antiviral effect of silymarin-associated drugs in chronic hepatitis C, liver transplantation, and difficult-to-treat HIV/HCV coinfected patients.

### 3.1. Chronic Hepatitis C

Several studies have evaluated the effect of silymarin or its component silibinin in patients with chronic hepatitis C. Oral administration of silymarin capsules ranging from 140 mg 3 times per day (for 1 year) to 700 mg 3 times per day (for 24 weeks) failed to decrease HCV viral load in three previous studies conducted in Egypt [33], Israel [34], and the United States [35]. However, interestingly, Malaguarnera et al. demonstrated in two randomized controlled trials (RCTs) that patients who received 12-month silybin-vitamin E-phospholipid complex pills supplemented to pegylated-interferon (Peg-IFN) + ribavirin (RBV) treatment achieved lower viral load compared to those who only received Peg-IFN+RBV treatment [36,37]. The silybin-vitamin E-phospholipid complex was reported to improve silybin’s solubility and bioavailability [38]; therefore, the difference between these results may imply the importance of improving silibinin or silybin’s solubility to enhance its effect in clinical use.

Consistent with the above results, intravenous infusion of Legalon^®^ SIL appeared to produce a better anti-HCV effect in clinical studies. Ferenci et al. demonstrated in a before-after study that 14 consecutive days of Legalon^®^ SIL infusion combined with 7 days of Peg-IFN+RBV treatment dose-dependently and continuously decreased HCV viral load in chronic hepatitis C patients who were previously non-responders to the Peg-IFN+RBV therapy [39]. After the infusion schedule, antiviral treatment with Peg-IFN+RBV and oral silymarin continued, and HCV RNA became undetectable in several patients of the 15 or 20 mg/kg/day groups. A later study analyzed the pattern in viral load decline over time with intravenous Legalon^®^ SIL monotherapy, and suggested that Legalon^®^ SIL may block both viral infection and viral production or release, with its dose-dependent effect mainly associated with blocking HCV production/release [40]. Following Ferenci’s study, Biermer et al. also reported a successful suppression of HCV viremia to undetectable level in a Peg-IFN+RBV non-responder patient using a combination of RBV, Legalon^®^ SIL (20 mg/kg/day), and Peg-IFN with a modified administration protocol [41]. The two groups also demonstrated in the next three years that additional Legalon^®^ SIL infusion, 20 mg/kg/day for 14 or 21 days [42], or 1400 mg/day for 2 days [43], could induce undetectable viral load in over half of the on-treatment non-responders to Peg-IFN+RBV therapy. Later in 2015, Dahari et al. published a case report showing that a patient who had severe adverse effect from IFN-containing regimen achieved SVR (sustained virological response) after 33 weeks of Legalon^®^ SIL infusion plus RBV and vitamin D therapy [44]. These results highlighted the potential of using Legalon^®^ SIL in combination with either IFN-based or IFN-free treatments to treat patients who are non-responsive or intolerable to IFN-containing treatments.

### 3.2. Liver Transplantation in Hepatitis C

HCV-associated liver cirrhosis and HCC are common indications for liver transplantation. The clearance of viremia pre-transplant or post-transplant is critical to prevent graft failure. However, with the introduction of DAAs for HCV, the need of transplantation has declined, and the management in liver transplant candidates and recipients has revolutionized [45]. The clinical trials reviewed here were conducted in the “pre-DAA era,” when IFN-based regimen remained the standard of care, and failure to achieve SVR with such regimen was a huge challenge for successful liver transplantation.

In 2010, Neumann et al. reported the first successful prevention of HCV reinfection and SVR 24 (sustained virological response in 24 weeks after treatment) after liver transplantation by the post-transplant administration of intravenous Legalon^®^ SIL monotherapy for 14 days in a genotype 3a patient who was non-responsive to Peg-IFN+RBV therapy [46]. The next year, Beinhardt et al. reported another IFN non-responder with mixed genotype 1a/4 infection achieving SVR 20 after liver transplantation by the administration of intravenous Legalon^®^ SIL monotherapy starting from 15 days pre-transplant to 25 days post-transplant [47]. Both groups suggested in their studies that relatively low viral load before transplantation could be a good prognostic factor [46,47]. Concordant with the viral load observation, Eurich et al. further reported a case series of four Peg-IFN+RBV non-responders who started intravenous Legalon^®^ SIL treatment months after liver transplant [48]. The patient who had the lowest viral load eliminated the virus during the first week of the 14-day Legalon^®^ SIL monotherapy, while the patient who had the second lowest viral load eliminated the virus under Peg-IFN+RBV therapy 2 months later. Both patients achieved SVR 24. As for the other two patients with higher initial viral load, 2.3 and 2.9 logs of viral load drop, respectively, were observed during the first 10 days of Legalon^®^ SIL administration, despite the rebounding viremia in the follow-up Peg-IFN+RBV therapy. On the other hand, Aghemo et al. presented a genotype 2a patient who started intravenous Legalon^®^ SIL monotherapy 24 h pre-transplant but failed to eliminate graft reinfection [49]. The authors proposed that this may be a result of genotype difference, but did not discuss about the relatively high viral load (more than 10^6^ IU/mL) before treatment of this patient [49]. In contrast, Knapstein et al. reported a genotype 3 Peg-IFN+RBV non-responder who also had pre-treatment viral load of more than 10^6^ IU/mL but successfully treated with Peg-IFN+RBV and intravenous Legalon^®^ SIL combination therapy at the post-transplant stage and achieved SVR 24 [50].

Later in 2013 and 2014, two randomized placebo-controlled trials [51,52] and a non-treated controlled trial [53] further explored the effect of intravenous Legalon^®^ SIL monotherapy to prevent HCV recurrence in liver transplantation setting. Both pre-transplant (a maximum of 21 consecutive days pre-transplant and 7 days post-transplant) [51] and post-transplant [52,53] administration of Legalon^®^ SIL significantly decreased the viral loads during the treatment; however, in all three studies, viremia rebounded after the end of treatment and became insignificant between the two groups, and no patient reached SVR after 24 weeks of follow up. These results confirmed the anti-HCV effect of Legalon^®^ SIL, but eradication of the virus may require longer administration or combination with other antivirals.

### 3.3. HIV/HCV Coinfection

Sharing similar transmission routes, 25–30% of HIV patients are coinfected with HCV [54]. Traditionally, HIV/HCV coinfected patients are known to be more difficult to treat compared to their mono-infected counterparts using Peg-IFN+RBV combination therapy. However, the use of the recently introduced DAAs in these patients has been demonstrated to yield similar SVR rates to those only infected with HCV [55]. Despite this miraculous achievement, the DAAs are very expensive and have the tendency to select for resistant mutants. Thus, finding alternative treatment strategies for the treatment of these patients is highly desirable. Due to the ability of silymarin and derivatives to target both viruses, several clinical trials have explored the use of these drugs in the HIV/HCV coinfected patients.

Payer et al. explored the use of Legalon^®^ SIL infusion in a 27-year-old female with the unfavorable single nucleotide polymorphism (SNP) IL28β genotype T/T, who was coinfected with HIV and HCV and refractory to Peg-IFN+RBV treatment [56]. The patient was subjected to Legalon^®^ SIL (20 mg/kg/day) monotherapy for 14 days. At day 8, the combination therapy with Peg-IFN+RBV was then started and continued until week 16 when the treatment had to be stopped due to psychiatric and other adverse events. Legalon^®^ SIL monotherapy for 1 week substantially decreased both HCV and HIV RNA levels, and after 2 weeks of Legalon^®^ SIL therapy including 1 week of Peg-IFN+RBV combination therapy, both HCV RNA and HIV RNA were undetectable. While the HIV RNA rebounded back 24 weeks after cessation of treatment, the HCV RNA remained negative during the same time frame [56].

Using a different protocol, Braun et al. examined the efficacy of Legalon^®^ SIL lead-in treatment in a total of 16 HIV/HCV coinfected Peg-IFN+RBV non-responding patients with advanced liver fibrosis in the clinical trial named THISTLE [57,58]. All patients were given 20 mg/kg/day of intravenous Legalon^®^ SIL monotherapy for 14 days, after which Peg-IFN+RBV combined with the HCV protease inhibitor Telaprevir was initiated for 12 weeks, followed by Peg-IFN+RBV dual regimen for another 36 weeks. Fifteen out of the 16 patients (94%) had undetectable HCV RNA at weeks 4 and 12, with 11 patients (69%) having undetectable HCV-RNA at week 48, and 10 patients (63%) reaching SVR at week 12. Six out of the 16 patients, however, could not achieve SVR 12. Collectively, these studies provide evidence that Legalon^®^ SIL lead-in treatment in combination with Peg-IFN+RBV and HCV protease inhibitors may be an exciting alternative to treat HIV/HCV coinfected patients to prevent potential drug–drug interactions and to improve treatment success with DAAs [58].

## 4. Challenges to Clinical Application and the Need to Enhance Bioavailability

Drug solubility has an important influence over drug absorption, and hence bioavailability. Despite the wide range of biological and pharmacological effects of silymarin, the extract is relatively insoluble in water (0.4 mg/mL), and the use of other solvents such as ethanol, glyceryl monooleate, polysorbate 20, and transcutol may help increase its solubility ranging from 33–350 mg/mL [59]. Studies based on silymarin’s primary active molecular component silybin indicate extensive enterohepatic circulation following oral administration, rapid excretion in bile and urine with an elimination half-life of about 6 h, and a low absorption from the gastrointestinal tract with a reported 0.73% of oral bioavailability in rat plasma [60,61]. In addition, the silybin content is particularly susceptible to conjugation reactions in phase II metabolism in the human liver, yielding various silybin metabolites conjugated with sulfates and glucuronides [61], and an observed average of 10% of the silybin isomers as unconjugated form in the plasma of orally-administered healthy volunteers [62]. The recent phase II trial demonstrated in chronic hepatitis C patients who received silymarin capsules that serum level of silybin varied significantly from 2.1 to 2048 ng/mL despite the high dose range used (420–700 mg, 3x daily) in the patient, indicating absorption and bioavailability issues which likely affected the efficacy outcome of the drug against hepatitis C [35]. The above factors contribute to the poor oral bioavailability of silymarin and, likewise, of its active constituent silybin. For this reason, most clinical trials and case studies, including those against chronic hepatitis C and HIV/HCV coinfection, employed the more water-soluble salt-derivatives such as Legalon^®^ SIL (silibinin-C-2′,3-dihydrogen succinate, disodium salt) [39,40,41,42,43,44,46,47,48,51,52,53,56,57,58]. However, Legalon^®^ SIL is inconvenient for administration, because it is given by *i.v.* infusion and cannot be administered orally. The available pharmacokinetic and clinical studies highlight the need to overcome drug delivery problems and formulate or modify silymarin and its active derivatives into more soluble forms that can achieve higher bioavailability.

To address this challenge, several methods have also been explored to increase the bioavailability of silymarin and its constituents. These include combination with phosphatidylcholine [63] or β-cyclodextrins [64], formation of salts and glycoside derivatives [65,66], liposome delivery [67,68], solid dispersion incorporation [69,70], self-microemulsifying drug delivery systems (SMEDDS) [59,71,72], and nanoformulations [73,74,75], which can all improve the solubility of silymarin as well as enhance the prolonged and sustained release of silybin. As an example, we have recently employed a nano-emulsification strategy in addressing the solubility and bioavailability issue of the standardized silibinin (silybin isomers). Specifically, silibinin-loaded nanoparticles (SB-NP) with diameters <200 nm were successfully developed using the hydrophilic carrier polyvinylpyrrolidone (PVP), which resulted in the transition of the silibinin crystalline structure into an amorphous state in the SB-NP and demonstrated a significantly enhanced solubility [15]. Interestingly, free silibinin was efficiently released from the nanoformulation at pH 7.4 but was prohibited at pH 1.2, indicating that the drug would be released extensively in the alkaline intestine rather than the acerbic stomach, thus favoring intestinal absorption. Importantly, the SB-NP retained their antioxidant activity and antiviral function against HCV infection in vitro, and were safe and orally bioavailable in vivo [15]. Enhanced serum concentration and superior biodistribution to the liver was observed compared to non-modified silibinin following oral administration in rats [15]. The orally applicable SB-NP with its improved solubility, absorption, and higher accumulation in the liver highlight an advantage for application against viral hepatitis, including hepatitis C, and underscores its potency for further development as a promising candidate drug agent.

Altogether, due to the widely known pharmacological effects but low solubility and bioavailability of silymarin and its derivatives, the above suggests that increasing the oral bioavailability is critical to their development and application in clinical settings. This is attested by the numerous studies to date, as mentioned above, aiming to address these challenges.

## 5. Prospects of Silymarin and Derivatives in Antiviral Development

A growing number of studies have demonstrated the hepatoprotective and antiviral effect of silymarin and derivatives both in vitro and in vivo. Although the in vivo hepatoprotective activity of the drug and its derivatives is ambiguous [76], partly due to the low bioavailability, improving the bioavailability for example through nano-formulation and other approaches could help solve this controversy. Various antiviral activities of silymarin and derivatives have been shown against liver and non-liver pathogens, making them potential broad-spectrum antivirals, at least for some of the enveloped viruses explored to date. In addition, considering the polypharmacological activity of silymarin and derivatives towards multiple host cell targets, such as cell innate immunity and inflammation [8,17], oxidative stress production [15], and autophagy [23], which are all cell physiological processes that are known to be elicited or subverted by many viral infections, these natural products are likely to exert their antiviral activities by modulating the cellular environment in addition to any potential direct antiviral function(s) against a specific viral protein. In the context of hepatic diseases, the ability of silymarin and derivatives to exert both hepatoprotective and antiviral activity makes them ideal candidates, particularly for hepatitis C with the greatest number of preclinical and clinical studies undertaken thus far. Given that the current antiviral agents, for example, the DAAs can only abrogate viral replication without displaying any hepatoprotective effects and are mechanistically different to silymarin’s known antiviral targeting activities, combining such drugs with silymarin or its derivatives would be expected to robustly improve the patient conditions. This notion is supported by the examples reviewed above, including the use of Legalon^®^ SIL in combination with PegIFN and/or RBV, or protease inhibitors in HCV non-responders [39,41,42,43,44,46,47,48,51,52,53,56,57,58]. These findings provide compelling evidence to explore the use of silymarin and derivatives in combination with existing antivirals as a potential treatment strategy, particularly for the treatment of chronic viral hepatitis. Further research to improve the bioavailability, delivery, as well as elucidating the main mechanism of antiviral activity of silymarin and derivatives could help to boost our understanding of these drugs and accelerate their development as hepatoprotective antiviral agents.

## Figures and Tables

**Figure 1:**
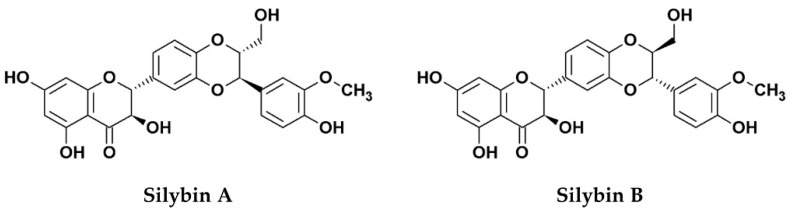
Chemical structures of silibinin, the 1:1 mixture of silybin A and silybin B.

**Table 1:** Preclinical studies of silymarin and derivatives in vitro and in silico.

Virus	Substrate(s)	Method(s)	Suggested Mechanism	Reference
Hepatitis C virus (HCV)	Silymarin extract (MK-001)	Western blot and RT-PCR	Potentiation of the JAK-STAT antiviral signaling pathway	[8]
	Silymarin and its-derived pure compounds	NS5B polymerase assay, luciferase reporter assay	Inhibition of HCV infection and the HCV-induced oxidative stress, as well as, the NS5B RdRp activity, NF-κB-dependent transcription, and T-cell receptor (TCR)-mediated proliferation	[9]
	Silymarin	NS5B polymerase assay, luciferase reporter assay, qPCR, and western blot	Inhibition of NS5B polymerase activity and blocking viral entry and transmission	[10]
	Silybin A, silybin B, and Legalon^®^ SIL	RdRp Enzyme Assay, qPCR and luciferase reporter activity	Inhibition of the NS5B RNA-dependent RNA polymerase	[11]
	Silibinin and Legalon^®^ SIL	HCV entry assay	Silibinin impeded HCV endosomal trafficking and blocked CME	[12]
	Silibinin	RT-PCR and luciferase reporter assay	Inhibition of HCV NS4B and hence the membranous web morphogenesis	[13]
	Silibinin nanoparticles	HCV entry assays and pharmacokinetic studies	Inhibition of HCV cell-to-cell spread and attenuation of HCV infection of PHHs	[15]
Dengue virus (DENV)	Silymarin	Docking to NS4B	All three silymarin derivatives docked with high binding affinity (≥−8 kal/mol) to DENV NS4B	[18]
Influenza A virus (IAV)	Silymarin	CPE reduction method	Inhibition of late viral RNA synthesis	[22]
	Silybin and amino acid derivatives (S0-S5)	CPE reduction method and plaque assay	S0 and S3 inhibited IAV replication and disrupted the formation of the Atg5-Atg12/Atg16L complex	[23]
Human immunodeficiency virus (HIV)	Legalon^®^ SIL	HIV replication in TZM-bl cells, peripheral blood mononuclear cells (PBMCs), and CEM	Attenuating cellular functions involved in T-cell activation, proliferation, and HIV infection	[26]
	Silibinin and Legalon^®^ SIL	HIV infection of PBMCs and CEM cells with respect to cell growth, ATP content, and metabolism	Perturbation of T-cell metabolism in vitro; Legalon^®^ SIL additionally blocked HIV infection of T-cells	[27]
Chikungunya virus (CHIKV)	Silymarin	CPE inhibition assay, RT-PCR and Western blot	Inhibition of CHIKV replication and proteinsynthesis	[28]
Mayaro virus (MAYV)	Silymarin	CPE inhibition, viral replication and plaque reduction assays in HepG2 cells	Inhibition of replication and ROS induction	[29]
Hepatitis B Virus (HBV)	Silibinin	HBV entry assay	Blockade of clathrin-mediated endocytosis	[31]

**Table 2:** Preclinical studies of silymarin and derivatives in vivo.

Virus	Substrate(s)	Analysis/Model	Route of Administration	Results	Reference
HCV	Legalon^®^ SIL	HCV infection of uPA-SCID-chimeric mice with humanized livers	Intravenous	Legalon^®^ SIL blocked HCV production and increased anti-inflammatory and anti-proliferative gene expressions without affecting serum albumin levels	[16]
IAV	Silybin derivatives (S0 and S3)	IAV infection of BALB/c mice	Oral	S0 and S3 increased the survival rate of mice (40% and 60% respectively), and S3 decreased virus titers in the lungs (100-fold)	[23]
HBV	Silymarin	HBV X protein (HBx) transgenic mice	Oral	Silymarin had no effect on HBx expression and late stage carcinogenesis, but recovered fatty acid change and liver pathology in the early stages of liver damage	[32]
